# Acute Responses of Low-Load Resistance Exercise with Blood Flow Restriction

**DOI:** 10.3390/jfmk9040254

**Published:** 2024-12-04

**Authors:** Andrew R. Jagim, Jordan Schuler, Elijah Szymanski, Chinguun Khurelbaatar, Makenna Carpenter, Jennifer B. Fields, Margaret T. Jones

**Affiliations:** 1 Sports Medicine, Mayo Clinic Health System, Onalaska, WI 54650, USA; 2 Department of Exercise and Sport Science, University of Wisconsin-La Crosse, La Crosse, WI 54601, USA; 3 Patriot Performance Laboratory, Frank Pettrone Center for Sports Performance, George Mason University, Fairfax, VA 22030, USA; jennifer.fields@uconn.edu (J.B.F.); mjones15@gmu.edu (M.T.J.); 4 Medical College of Wisconsin-Central Wisconsin, Wausau, WI 54401, USA; 5 Department of Nutritional Sciences, University of Connecticut, Storrs, CT 06269, USA; 6 Sport, Recreation, and Tourism Management, George Mason University, Fairfax, VA 22030, USA

**Keywords:** blood flow restriction, strength training, physiological response, lower body power, recovery, metabolic stress

## Abstract

Blood flow restriction (BFR) is a popular resistance exercise technique purported to increase metabolic stress and augment training adaptations over time. However, short-term use may lead to acute neuromuscular fatigue and higher exertion ratings. **Objective:** The purpose of the current study was to examine acute physiological responses to low-load resistance exercise utilizing BFR compared to higher-load, non-BFR resistance exercise. **Methods:** Recreationally trained males (n = 6) and females (n = 7) (mean ± standard deviation, age: 20 ± 1 yrs.; height: 172 ± 8 cm; weight: 73 ± 11 kg; BMI: 24.4 ± 2.2 kg·m^−2^; training experience: 4 ± 2 yrs.) had limb occlusion pressure determined (50%; right leg: 118 ± 11 mmHg; left leg: 121 ± 13 mmHg) using an automated, self-inflating cuff system during baseline testing. In subsequent sessions, using a randomized, cross-over design, participants completed one of two experimental conditions: (1) Low-load + BFR and (2) High load + non-BFR. In both conditions, participants completed one set of back squats at either 30% (BFR) or 60% (non-BFR) of an estimated 1RM for a max of 30 repetitions, followed by three additional sets with the same loads and a target of 15 repetitions per set. Blood lactate and countermovement jump (CMJ) height were measured pre- and post-back squat. Ratings of perceived exertion (RPE) were assessed following each set. **Results:** When collapsed across all sets, participants completed significantly more total repetitions in the BFR condition compared to non-BFR (75.0 ± 0.0 vs. 68.23 ± 9.27 reps; *p* = 0.015; ES: 1.03), but a lower training load volume (2380 ± 728 vs. 4756 ± 1538 kg; *p* < 0.001; ES: 1.97). There was a significant time-by-condition interaction (*p* < 0.001), with a greater increase in blood lactate occurring from baseline to post-back squat in the non-BFR condition (11.61 mmol/L, 95%CI: 9.93, 13.28 mmol/L) compared to BFR (5.98 mmol/L, 95%CI: 4.30, 7.65 mmol/L). There was another significant time-by-condition interaction (*p* = 0.043), with a greater reduction in CMJ occurring in the non-BFR condition (−6.01, 95%CI: −9.14, −2.88 cm; *p* < 0.001) compared to BFR (−1.50, 95%CI: −1.50, 4.51 cm; *p* = 0.312). **Conclusions:** Utilizing a low-load BFR protocol may allow for a higher training volume, yet lower metabolic stress and reduce neuromuscular fatigue compared to lifting at a higher load without the use of BFR.

## 1. Introduction

Regular resistance training is well known to increase muscular strength and hypertrophy for a variety of populations and can even play a role in clinical settings to recover muscle functionality and reduce risk factors for sarcopenia [1,2,3,4]. With a goal of muscle hypertrophy, the most common training strategy is the utilization of consistent resistance training, with moderate-to-high-load training equating to ≥60–75% of one-repetition maximum (1RM), completed to near failure, while incorporating progressive overloading strategies over time [5,6,7]. However, several advanced training techniques are often utilized to further augment acute hypertrophic signaling mechanisms. One such strategy gaining recent popularity is the use of blood flow restriction (BFR), a technique often used in conjunction with resistance training to further elicit metabolic stress and enhance anabolic signaling mechanisms [8,9]. Blood flow restriction has applications for a variety of populations including athletes [10], older adults [11], and clinical populations [12]. The use of BFR appears to enhance intramuscular metabolic stress and augment anabolic signaling mechanisms by restricting local venous return to the muscle groups involved in the designated activity or specific exercise [13,14,15]. Research has indicated that using lighter loads during resistance training with BFR may provide equivocal benefits to resistance training without BFR at higher relative loads, while also reducing the risk of injury [13,16,17].

In order to develop evidence-based protocols for BFR applications, it is important to continue to examine the acute physiological responses when using BFR as part of a resistance training protocol to establish best practices and guidance for use. BFR is most commonly performed by utilizing a device such as a cuff or a tourniquet that occludes venous return, which lessens the need for high-load training and subsequently reduces the mechanical tension required to elicit a similar anabolic response [18,19]. Specifically, a limb occlusion pressure of 50% (half of the external pressure required to occlude venous blood flow) has been shown to result in restricted blood flow and has been determined to be the optimal pressure to use in practice [15,20]. However, higher occlusion pressures across various training intensities have also been used and found to be beneficial when used in conjunction with resistance exercise [10,16,21,22]. In turn, this occlusion pressure appears to subsequently influence muscle activation patterns, induce temporary ischemia, and result in temporary sensations of pain during resistance training activities [15,22,23,24,25]. The primary mechanism of action is thought to be a result of decreased intramuscular oxygen availability and the accumulation of metabolites within the muscle while venous blood flow is occluded [24,26,27]. The exact mechanism for how lower-intensity BFR stimulates cellular processes to induce comparable or even superior muscle hypertrophy to non-BFR high-intensity methods has not been fully elucidated. Several hypotheses have suggested that the enhanced production of cellular metabolites and growth hormone may play a role, in addition to the increase in cellular edema commonly reported during BFR training [9,23,27,28].

Recent evidence has indicated that muscle hypertrophy can occur across a spectrum of load intensities if sufficient total training volume is accrued (e.g., the number of total repetitions) [6]. A purported advantage of using BFR is that there may be the ability to elicit comparable acute physiological responses from using low loads (e.g., <50% of 1RM) with BFR compared to higher loads without BFR. A recent study found that the use of BFR during multijoint resistance training exercises elicits a similar increase in hormonal responses during low load (20% 1RM) + BFR, compared to high load (75% 1RM) resistance exercise without BFR. However, there are concerns of increased exercise-induced muscle damage following BFR that have been raised as a byproduct of the increased metabolic stress being imposed [29]. However, previous research has found that low-load resistance training with BFR leads to significant increases in muscle activation without increasing indices of muscle damage following lower body resistance exercise compared to non-BFR training in resistance-trained males [26]. Additionally, previous research has found that acute metabolic stress, as measured by blood lactate accumulation, can serve as a mechanism of muscle hypertrophy, and therefore examining which combination of exercise loading and BFR elicits the largest response in blood lactate accumulation may be important from a exercise prescription standpoint [7,30,31].

Further, it is important to examine the acute performance effects of resistance exercise with BFR to better understand how and when BFR should be implemented within an exercise session or throughout the day if there is a need to mitigate fatigue. Various performance and laboratory measures have been used to evaluate neuromuscular fatigue and metabolic stress. For example, countermovement jump (CMJ) height before and after exercise may be used as an indicator of exercise-induced neuromuscular fatigue and monitor changes in lower body explosiveness [32,33,34]. Further, ratings of perceived exertion can be used as self-reported indicators of muscular loading [35,36,37]. The use of these assessments can provide insight into the acute effects of resistance exercise with BFR; however, current research exploring the impact of BFR on measures of metabolic indices (i.e., blood lactate response), neuromuscular fatigue, and self-perceived effort following resistance exercise is limited. This information could help provide insight into how acute resistance exercise protocols using BFR may influence short-term measures of metabolic stress, fatigue, and subjective measures of exertion and help determine how or when BFR interventions could be used in a strength and conditioning program.

There is a high demand for methods that mitigate the risks associated with resistance training, while not compromising the potential to stimulate muscle hypertrophy [17]. Therefore, BFR has applications in both performance-based and clinical settings, as well as for the general public with the goal of augmenting muscular hypertrophy, while reducing risks associated with high-load training (>85% 1RM) [9,13]. There continues to be a need to further explore the acute physiological responses of low-load resistance training in conjunction with BFR compared to higher-load resistance training. Moreover, it is important to understand the short-term effects on target repetitions and subsequent effects on physical performance, metabolic stress, and the perception of effort when using BFR during resistance training activities. To the best of our knowledge, no study has examined the differences in low-load resistance training with BFR to high-load non-BFR training and the resulting effects on training load-volume, lactate, power, and perceptions of effort. Therefore, the purpose of this study was to examine acute responses to low-load resistance exercise utilizing BFR compared to higher-load, non-BFR training. The primary outcome measures included training volume and blood lactate responses. Secondary outcome measures included neuromuscular fatigue (as determined thought vertical jump assessment) and ratings of perceived exertion.

## 2. Materials and Methods

### 2.1. Study Design

**Figure 1:**
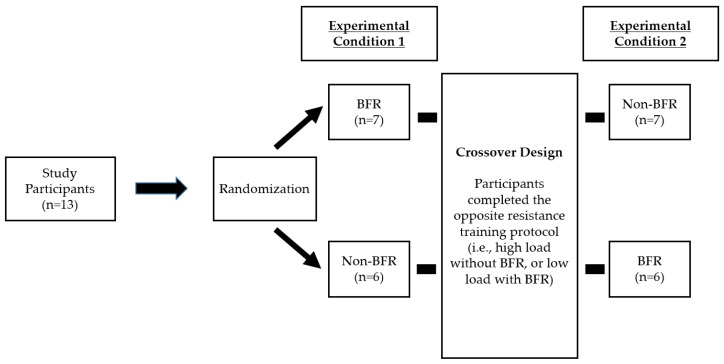
Consort diagram.

Using a randomized, cross-over design, participants completed one of two experimental conditions: low load (30% 1RM) + BFR; or high load (60% 1RM) + non-BFR. Heart rate, blood pressure, blood lactate, and countermovement vertical jump (CMVJ) height were measured pre- and post-back squat for each respective condition. Ratings of perceived exertion (RPE) were assessed following each set. Within 7 days, participants returned to the lab to complete the opposite condition in a randomized fashion (randomizer.org) while following the same testing protocol. Figure 1 presents a summary of the consort diagram.

### 2.2. Subjects

Thirteen recreationally trained college-aged males (n = 6) and females (n = 7), (mean ± standard deviation, age: 19.6 ± 1.2 yrs.; height: 171.9 ± 7.7 cm; weight: 72.5 ± 10.8 kg; BMI: 24.4 ± 2.2 kg·m^−2^) participated in the current study. Participants had an average of 3.8 ± 2.5 yrs. of resistance training experience, with an average relative back squat 1RM of 1.5 ± 3.1 times their bodyweight. Participants were recruited from a local university via word of mouth and recruitment flyers. All interested participants first completed the physical activity and readiness questionnaire (PAR-Q) to identify any potential contraindications to exercise. Inclusion criteria included being a recreationally active male or female between the ages of 18 and 27 with >1 year of consistent resistance training (>2 times per week). Exclusion criteria included having cardiovascular disease, a recent lower body injury (within the past 3 months), a history of clotting disorders or strokes, or being on any medications that may influence clotting or blood viscosity. Participants were then informed of the details of study participation and provided written consent approved by the University’s Institutional Review Board (IRB Protocol: 22-JS-12; Approved on: 29 July 2021), and all study procedures were conducted according to the Declaration of Helsinki and Human Subjects Research Guidelines. The clinical trial was registered through the International Standard Randomized Controlled Trial Number Registry (ISRCTN28808565).

### 2.3. Baseline Testing

**Figure 2:**
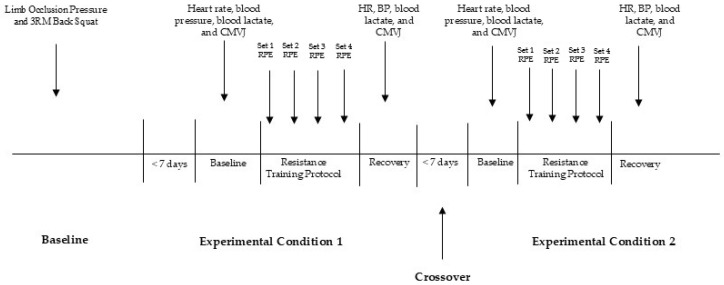
Summary of testing procedures and timelines.

During initial baseline testing, participants first had limb occlusion pressure (50%) determined for each limb. Following limb occlusion determination, a 1RM back squat was estimated from a 3RM test [38] using a Smith machine (Plyometric Power System; Norsearch, Australia). Prior to the 3RM assessment, all participants completed a standardized warm-up, consisting of dynamic whole-body stretches which lasted approximately 5–10 min. Participants then completed a warm-up set of ten repetitions, using a load that corresponded to an estimate of 50% of their 3RM. Participants then completed a second warm-up set of 6 repetitions at a load corresponding to approximately 70% of their perceived 3RM with two minutes of rest provided in between each set. Participants then completed maximal effort sets to determine 3RM, with progressively increasing loads until a 3RM was determined within three to five 3RM attempts and two minutes of rest provided between each attempt. Following 3RM assessment, participants were familiarized to the back squat protocol and BFR cuffs to help them get accustomed to the protocol and feeling of BFR during resistance training. Figure 2 provides a summary of testing procedures.

#### 2.3.1. Hemodynamics

Resting heart rate and blood pressure were initially assessed at baseline using standard clinical assessment guidelines. Heart rate was manually palpated at the radial artery for a 60 s count prior to and immediately post-back squat. Blood pressure was assessed with the participants seated in an upright position after a resting period of 5 min, with the exception of post-back squat assessments, which were conducted immediately post-back squat (<60 s post). A mercurial sphygmomanometer (American Diagnostic Corporation, model #AD-720) was used according to standard procedures [39]. Blood pressure and heart rate were also evaluated immediately post-inflation of the BFR cuffs for the BFR condition only.

#### 2.3.2. Blood Lactate and Ratings of Perceived Exertion

Blood samples were taken from the fingertips at baseline and five minutes into the recovery phase post-back squat to assess whole blood lactate levels. Lactate was determined using a Lactate Scout (Sports Resource Group, Edina, MN, USA) handheld analysis device. Previous research has yielded a mean intraclass correlation of 0.91 and a mean intraclass coefficient of variation of 10.2% [40]. Calibration procedures were completed prior to each testing session. Subjects were asked to record their rating of perceived exertion using Borg’s 15-point scale [41] following each set.

#### 2.3.3. Neuromuscular Fatigue

A countermovement vertical jump test was used to evaluate changes in neuromuscular fatigue. Subjects were instructed to jump as explosively as possible with their hands on their hips. A 27″ × 27″ jump mat (Just Jump System, Probotics, Huntsville, AL, USA) was used to record jump height derived from flight time which was instantaneously calculated and presented on a digital display. The jump mat has previously been shown to be strongly correlated to criterion measures of flight time (r = 0.969) and jump height (r = 0.972) using a force plate [42]. The countermovement vertical jump (CMJ) was completed at baseline, prior to the back squat protocol, and immediately following hemodynamic measurements post-back squat.

#### 2.3.4. Resistance Training Protocol

In both conditions, participants completed 1 set of 30 repetitions following the respective resistance training protocol, at the assigned load. Participants then completed 3 sets with a maximum number of 15 repetitions allowed in sets 2–4, with a 2 min rest in between sets similar to previous methods [26].

#### 2.3.5. Blood Flow Restriction

During baseline testing, participants had limb occlusion pressure (50%) determined using an automated, self-inflating cuff system (SmartCuffs©, Smart Tools Plus, LLC, Strongsville, OH, USA). During the experimental BFR condition, the cuff was inflated to the determined leg-specific pressure, set at 50% of total limb occlusion pressure (right leg: 118 ± 11 mmHg; left leg: 121 ± 13 mmHg) and left on for the duration of the protocol, including rest periods. Table 1 provides an outline of the resistance training protocol for each experimental condition.

## 3. Statistical Analysis

A within-subjects, repeated-measures analysis of variance was used to evaluate differences in repetitions completed and ratings of perceived exertion for each set. A within-subjects, repeated-measures analysis of variance was used to evaluate differences in blood lactate, countermovement vertical jump height, and hemodynamic variables before and after the back squat exercise for each condition. An a priori power analysis using G*Power (Version 3.1) with estimates of effect sizes from previous studies examining acute physiological responses during exercise with BFR was conducted. The most conservative effect size indicated that a sample size of 10 subjects for each condition would be sufficient to detect clinically meaningful differences indicative of an effect size of 0.6 (moderate) for the number of repetitions completed, using a two-tailed test (alpha = 0.05) with 80% power based on previous findings [43,44,45]. Data are presented as mean ± standard deviation. The statistical significance was set at *p* < 0.05. Effect sizes (Cohen’s d) were reported, where appropriate, and interpreted as follows: large (d > 0.8), moderate (d = 0.8–0.5), small (d = 0.49–0.20), and trivial (d < 0.2) [46]. Normality was initially assessed using the Shapiro–Wilk test and homoscedasticity was assessed with Levene’s Test of Equality of Error Variances. All data were analyzed using the Statistical Package for the Social Sciences (IBM SPSS Statistics for Windows, Version 26.0: IBM Corp., Armonk, NY, USA).

## 4. Results

### 4.1. Training Volume

Participants completed overall more repetitions of the back squat in the BFR condition compared to non-BFR (BFR: 75.0 ± 0.0 vs. non-BFR: 68.2 ± 9.3 reps; *p* = 0.015; ES: 1.03). There was a lower training load volume in the BFR condition compared to the non-BFR condition (2380 ± 728 vs. 4756 ± 1538 kg; *p* < 0.001; ES: 1.97).

### 4.2. Blood Lactate

**Figure 3:**
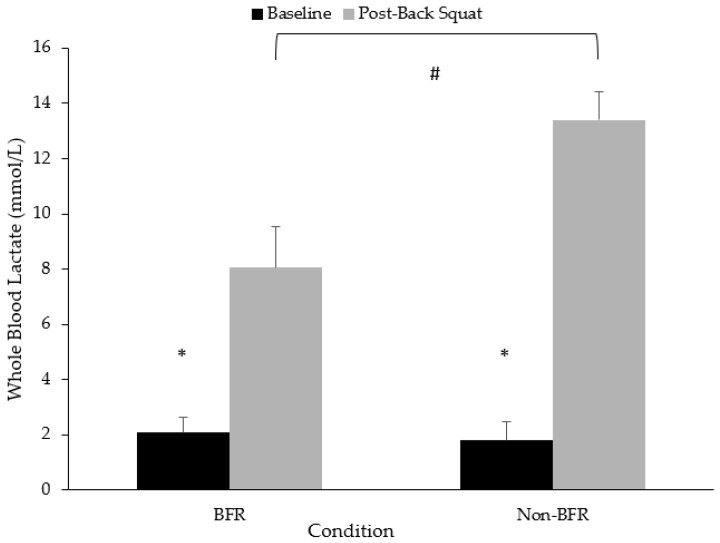
Changes in blood lactate values across conditions from pre- to post-back squat. * Denotes significant difference from baseline to post-back squat (*p* < 0.05); # Denotes significant group x time interaction (*p* < 0.05).

There was a significantly greater increase in blood lactate from baseline to post-back squat in the non-BFR condition compared to the BFR condition (11.6 mmol·L^−1^, 95%CI: 9.9, 13.3 mmol·L^−1^ vs. 5.9 mmol·L^−1^, 95%CI: 4.3, 7.7 mmol·L^−1^) (Figure 3).

### 4.3. Ratings of Perceived Exertion

**Figure 4:**
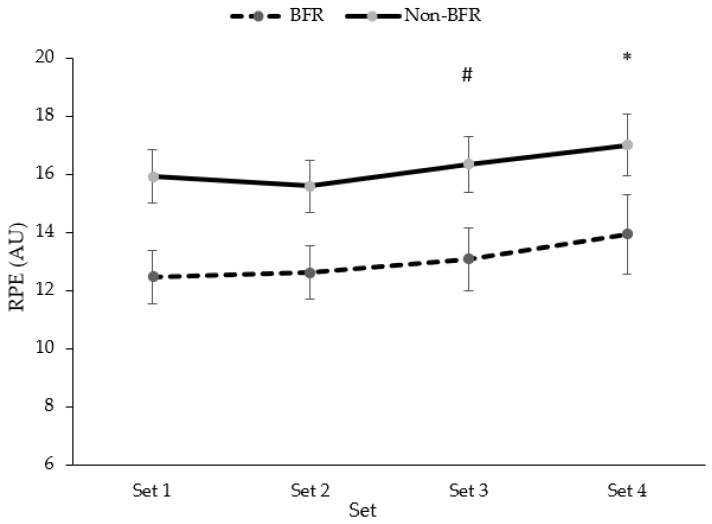
Changes in ratings of perceived exertion across sets 1–4 of the back squat exercise. * Denotes significant difference from baseline (*p* < 0.05); # Denotes significant group x time interaction (*p* < 0.05).

There was a main effect for sets observed for RPE (*p* = 0.009), but no significant set-by-condition interaction (*p* = 0.747) (Figure 4).

### 4.4. Countermovement Vertical Jump

**Figure 5:**
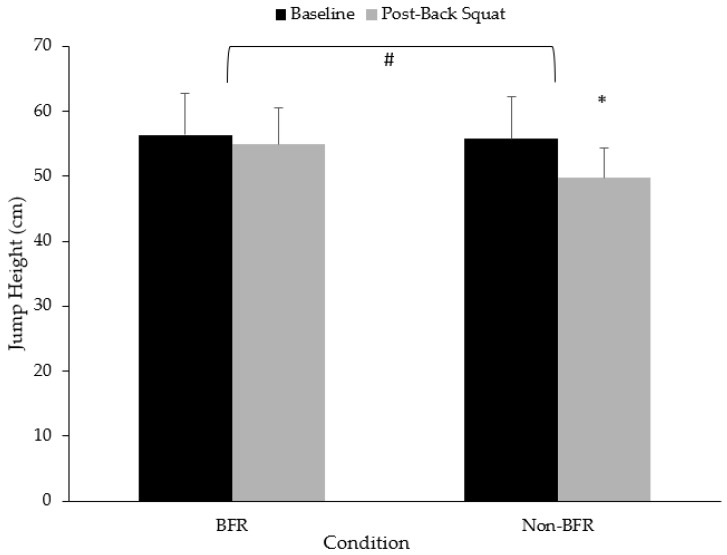
Changes in countermovement vertical jump height from pre- to post-back squat. * Denotes significant difference from baseline to post-back squat (*p* < 0.05); # Denotes significant group x time interaction (*p* < 0.05).

There was a significantly greater reduction in the CMJ height in the non-BFR condition (−6.0, 95%CI: −9.1, −2.9 cm; *p* < 0.001) compared to BFR (−1.5, 95%CI: −1.5, 4.5 cm; *p* = 0.312) (Figure 5).

### 4.5. Hemodynamic Response

A summary of the hemodynamic responses across each condition is presented in Table 2. No significant changes in heart rate systolic blood pressure (SBP) or diastolic blood pressure (DBP) occurred from baseline to immediate post-BFR application (*p* > 0.05). There was a significant time-by-condition effect observed for SBP (*p* = 0.005). Pairwise comparisons indicated that the non-BFR condition resulted in a larger decrease in SBP from baseline to post-back squat (−16.5, 95%CI: −22.4, −10.6 mmHg; *p* < 0.001) compared to the BFR condition (−4.0, 95%CI: −9.9, −1.9 mmHg; *p* = 0.175). There was a significant time-by-condition effect observed for DBP (*p* = 0.002). Pairwise comparisons indicated that the non-BFR condition resulted in a larger decrease in SBP from baseline to post-back squat (−11.7, 95%CI: −19.2, −4.2 mmHg; *p* = 0.004) compared to the BFR condition (−6.6, 95%CI: −14.1, 0.9 mmHg; *p* = 0.08).

## 5. Discussion

The main findings from the current study indicate that completing four sets of the back squat at 30% of 1RM with BFR resulted in lower blood lactate levels and lower RPE values post-back squat, despite more total repetitions across the four sets completed in the BFR condition compared to non-BFR (performed at 60% 1RM). Further, a lower degree of neuromuscular fatigue was observed following the back squat with low load (30% 1RM) with BFR compared to high load (60% 1RM) without BFR. The results of the current study indicate that BFR training may allow participants to achieve a higher training volume while resulting in less metabolic stress compared to higher-load non-BFR training. However, training load volume was higher in the non-BFR condition, because of the higher relative load used.

In the current study, the higher number repetitions completed in the BFR condition was to be expected as a result of the lower load being used (30% 1RM vs. 60% 1RM); however, this is in contrast to previous reports and appears to be influenced by the specific resistance exercise protocol used. For example, in a study by Hornikel et al. [47], resistance-trained males completed fewer repetitions when completing four sets of back squats to failure at 75% 1RM when using BFR, compared to the non-BFR control condition. This is likely due to heavier loads being used, and equivalent loads across the BFR and non-BFR conditions in comparison to the current study’s protocol (30% 1RM and 60% 1RM). In a similar study by Loenneke et al. [48], participants completed a set of leg extensions to failure at 30% of 1RM, and it was reported that the addition of BFR resulted in lower repetitions. Interestingly, lower blood lactate values were also observed in the BFR condition immediately after muscular failure. However, in the current study, participants completed more repetitions with the use of a lower load with BFR across four sets, yet it resulted in lower blood lactate values. It is possible that the higher load used in our control condition (60% 1RM), contributed to a greater overall training load volume and thus a higher degree of metabolic stress.

The results from the current study also indicate that low-load BFR training may serve as a way to mitigate short-term decrements in neuromuscular fatigue post-resistance training compared to higher load non-BFR training. Specifically, the low-load BFR condition resulted in a reduction of 1.5 cm compared to a reduction of 6 cm in the higher-load non-BFR condition. It is worth noting that this observation may also be explained by differences in the volume of work completed, as discussed in the sections above. Previous studies have shown that BFR training can increase muscle activation and contribute to increases in force production and bar velocity in acute settings [26,49,50]. A study by Wilk et al. [49] found that bar velocity was higher during multiple sets of bench press when performed with BFR compared to a non-BFR condition at 70% of 1RM. Specifically, they found greater peak and mean power, and greater peak and mean velocity across multiple sets of bench press when completed with BFR. Serrano-Ramon also observed improvements in intraset bar velocity during the back squat at 90% 1RM [50]. While conducted after the back squat exercises, results from the current study indicated a better maintenance of CMJ height post-back squat with BFR (using a lighter load) compared to the non-BFR condition, indicating that higher-load non-BFR training results in greater neuromuscular fatigue as opposed to BFR training.

In the current study, the use of BFR with lower relative loads resulted in lower RPE values throughout the four sets of back squats. Previous research has found mixed effects of using BFR on perceptions of effort and discomfort during exercise. For example, Bell et al. [51] observed increased ratings of perceived exertion across low-load BFR conditions (15% of 1RM at 80%, 40%, and 0% occlusion) when completed to failure compared to a high-load (70% of 1RM) condition during a unilateral elbow flexion exercise. Furthermore, exercise volume was found to be similar for the low-load BFR conditions with 0% and 50% arterial occlusion pressure at 15% of 1RM, but significantly higher than the BFR condition with 80% occlusion and the no-BFR high-load condition at 70% of 1RM in the study by Bell et al. [51]. There is also evidence of low-load (30% of 1RM) resistance exercise with BFR leading to a greater degree of hypoalgesia when compared to high-load resistance exercise (70% of 1RM), as observed by Norbury et al. [52], suggesting that while low-load BFR resistance exercise may be perceived as more difficult, it could provide a greater hypoalgesic effect. Additionally, the addition of BFR has been shown to lead to a greater perception of effort, despite greater tissue oxygen saturation during high-intensity cycling exercise, as observed by Lauver et al. [43]. Additionally, in the study by Lauver et al. [43], the acute increase in perception of effort coincided with a greater rating of discomfort as the perception of effort was significantly greater in BFR conditions as opposed to non-BFR conditions. However, the study by Lauver et al. [43] did not further investigate changes in metabolic stress or hemodynamic responses to aerobic training in the BFR and non-BFR conditions. Investigation into these physiological responses such as blood lactate, would further clarify the potential implications of BFR use in high-intensity aerobic exercise. It appears that the influence of BFR on RPE is largely due to the relative load being used in addition to the target training volume.

More research is needed in monitoring BFR protocols during complete workouts, rather than isolated exercises. Additionally, comparing the cellular mechanism of BFR training that induces comparable or even superior improvements in muscle strength and hypertrophy without increasing potential markers of muscle damage could be explored. This would allow for greater understanding and evidence as to how participants can use BFR to increase strength and muscle hypertrophy without increasing physiological and psychological risks associated with exercise. Furthermore, a longitudinal study investigating the impacts of combining BFR and non-BFR training on muscle hypertrophy and participant performance could yield more practical implications of BFR in athlete training regiments. There is a wide market in therapeutics and athletic performance to optimize and largely implement BFR training methods into practice.

## 6. Limitations

This study is not without limitations. The presence of a third condition (low load without BFR) would have allowed for additional insight regarding how the addition of BFR affects the physiological responses to low-load resistance training. Further, an additional timepoint at 24 h post-testing would have provided information regarding the effects of BFR on muscle soreness and the potential onset of delayed fatigue. Lastly, the failure to include electromyography analysis is another limitation of the current study design that would have helped identify differences in muscle activation patterns between the two conditions. The small sample size also limited the ability to examine sex differences in response to BFR training. Additionally, the young age of the study participants may limit the transferability of the study findings to older adult or clinical populations.

## 7. Conclusions

Completing four sets of the back squat at 60% of 1RM without BFR resulted in higher blood lactate levels and RPE post-exercise, even though fewer repetitions were performed compared to the BFR condition (completed with 30% 1RM). Using a low-load BFR protocol (30% of 1RM) may allow for a higher training volume, yet lower metabolic stress and fewer detrimental effects on neuromuscular performance compared to lifting at a higher load (60% of 1RM) without BFR. When utilizing BFR with a lighter relative load, individuals may be able to mitigate short-term metabolic stress and neuromuscular fatigue if that is a concern. While in the low-load BFR condition, participants were able to complete a higher number of total repetitions, the overall training load volume was much lower in the low-load BFR condition. Therefore, if maximal strength development is a primary training goal, the higher training load (without BFR) would likely be more beneficial.

## Figures and Tables

**Table 1:** Resistance training protocols for BFR and non-BFR conditions.

Set	Condition	
	BFR	Non-BFR
1	30 × repetitions at 30% of the individuals’ 1RM (with BFR)	30 × repetitions at 60% of the individuals’ 1RM (without BFR)
2	15 × repetitions at 30% of the individuals’ 1RM (with BFR)	15 × repetitions at 60% of the individuals’ 1RM (without BFR)
3	15 × repetitions at 30% of the individuals’ 1RM (with BFR)	15 × repetitions at 60% of the individuals’ 1RM (without BFR)
4	15 × repetitions at 30% of the individual’s 1RM (with BFR)	15 × repetitions at 60% of the individuals’ 1RM (without BFR)

1RM = one-repetition maximum; BFR = blood flow restriction.

**Table 2:** Summary of hemodynamic responses for each condition.

	HR		SBP		DBP	
	Pre	Post	Pre	Post	Pre	Post
Non-BFR (n = 13)	74 ± 14	147 ± 18	115 ± 5	131 ± 9	73 ± 6	61 ± 13
BFR (n = 13)	78 ± 13	135 ± 26	120 ± 9	124 ± 11	69 ± 10	76 ± 12

Data presented as means ± standard deviation; HR = heart rate; SBP = systolic blood pressure; DBP = diastolic blood pressure.

## Data Availability

Data are available upon request.
